# Anticonvulsants for behavioral and psychological symptoms in dementia: protocol for a systematic review

**DOI:** 10.1186/s13643-019-1025-5

**Published:** 2019-05-18

**Authors:** Sophiya Benjamin, John W. Williams, Cecilia Cotton, Jennifer Tung, Howard An, Stephanie Sanger, Joanne Man-Wai Ho

**Affiliations:** 1 Department of Psychiatry & Behavioral Neurosciences, McMaster University, 10b Victoria St S, Kitchener, ON N2G 1C5 Canada; 2 Duke University and the Durham Veteran Affairs Medical Center, Durham, NC USA; 3 University of Waterloo, Waterloo, ON Canada; 4 Grand River Hospital, Kitchener, ON Canada; 5 Toxicology, Trillium Health Partners - Credit Valley Hospital, Mississauga, ON Canada; 6 McMaster University, Hamilton, ON Canada; 7 Department of Medicine, McMaster University, Hamilton, ON Canada; 8 Schlegel-UW Research Institute for Aging, Waterloo, ON Canada

**Keywords:** Dementia, Major neurocognitive disorder, Agitation, Behavioral and psychological symptoms, Neuropsychiatric symptoms, Anticonvulsants

## Abstract

**Background:**

Behavioral and psychological symptoms of dementia (BPSD) are present in a majority of patients with dementia contributing to increased morbidity, health care costs, and caregiver burden. While there are no United States Food and Drug Administration (FDA)-approved medications for these symptoms, off-label use of medications such as antipsychotics have been shown to have significant adverse effects including increased mortality. The goal of this review is to examine the efficacy and safety of anticonvulsants in the treatment of BPSD.

**Methods:**

We will systematically search for randomized trials of anticonvulsants compared to placebo or other treatments such as antidepressants and antipsychotics from the following sources: The Cochrane Library, MEDLINE (OVID SP) in Process and Other Non-Indexed Citations (latest version), EMBASE, clinicalTrials.gov, and the WHO Clinical Trials Registry. The studies will be limited to those published in English but the study location can be worldwide. We will include studies pertaining to individuals with dementia and symptoms of BPSD. The primary outcomes will be behavioral change as measured by validated scales and secondary outcomes will include caregiver burden, quality of life, placement in long term care facility, serious adverse effects, and treatment discontinuation due to adverse effects. Two sets of reviewers will independently screen select and extract data. We will narratively describe the major findings and conclusions from individual studies. Patients who are prescribed antiepileptic drugs (AEDs) for other indications, including seizures, will be excluded. Outcomes of interest will include a change in a validated scale that measures BPSD, serious adverse events, and caregiver quality of life outcomes. If the data are found to be appropriate for a meta-analysis, we will use a random effects model to compute summary estimates of treatment effects.

**Discussion:**

This is a protocol for a systematic review addressing the anticonvulsant group of medications as a whole, and as such, our results will inform current clinical practice in the use of anticonvulsants for BPSD. It will also help clinicians and policy makers compare the efficacy of anticonvulsants compared to antidepressants and antipsychotics as well as identify areas which will need further study.

**Systematic review registration:**

PROSPERO CRD42017079826

**Electronic supplementary material:**

The online version of this article (10.1186/s13643-019-1025-5) contains supplementary material, which is available to authorized users.

## Background

Behavioral and psychological symptoms of dementia (BPSD) can present as disturbances in mood, perception, or behavior in a majority of individuals with dementia. These behaviors can lead to caregiver burnout, nursing home placement [1], and increase the cost of care in individuals with dementia estimated at about $1298/month [2].

Of the pharmacological agents used to treat BPSD, antipsychotics have the largest number of studies conducted; however, the effects of these antipsychotics in treating these symptoms are small. Recent studies about the side effects of antipsychotics in dementia estimate that the risk of increased mortality in patients with dementia is higher than previously estimated [3]. Antidepressants have also been studied in the treatment of BPSD with evidence both from RCTs [4] and systematic reviews [5, 6]. A more recent systematic review did not show strong support for antidepressants in the treatment of depressive symptoms in dementia [7]. There continues to be a need for other agents as not all patients respond to antipsychotics and SSRIs. The effects of anticonvulsants which are also used in mood disorders have been studied as potential treatments for BPSD; however, the effectiveness and risks of this class of medications are yet to be proven.

Behavioral and psychological symptoms in dementia are largely thought to be multifactorial and result from the interplay between neurobiological and precipitating environmental factors [8]. Although the exact means by which anticonvulsants affect these symptoms are unknown, these agents directly affect neuronal excitability by increasing GABAergic inhibitory neurotransmission, decreasing glutamatergic neurotransmission, inhibiting voltage-dependent sodium or calcium channels, and impacting intracellular signaling pathways [9]. Specifically, carbamazepine inhibits voltage-dependent sodium and calcium L-type channels, decreases glutaminergic transmission, and blocks adenosinergic receptors [8, 10]. Its derivative, oxcarbazepine, works similarly [10]. Valproate, a broad spectrum drug, increases GABAergic transmission and inhibits voltage-dependent sodium channels [11]. It also affects intracellular pathways and gene expression, including inhibition of histone deacetylases (HDACs) [11]. Lamotrigine resembles carbamazepine and valproate in its mechanism by inhibiting voltage-dependent sodium and calcium channels [12]. Topiramate inhibits voltage-dependent sodium and calcium channels, enhances GABAergic activity, and antagonizes AMPA/kainite receptors [13]. Levetiracetam binds to synaptic vesical protein SV2A, although its exact mechanism of action has yet to be fully elucidated [8]. Gabapentin and pregabalin modulate postsynaptic excitotoxicity primarily by binding to the α_2_δ subunits of presynaptic voltage-dependent calcium channels [13].

With all antipsychotics (with the exception of risperidone in Canada in limited circumstances) being off-label in the treatment of BPSD and the new FDA and Health Canada warnings about citalopram and escitalopram, anticonvulsants have received more attention and interest as alternate agents in the treatment of these difficult and distressing symptoms in dementia. There are several descriptive reviews on the topic of anticonvulsants in BPSD [8, 14, 15] and a Cochrane review focusing on valproic acid that was completed in 2004 [16] and repeated in 2009 [17] and 2018 [18]; however, to the best of our knowledge, there have been no systematic reviews examining the current evidence on the entire group of antiepileptic drugs (AEDs) in the treatment of BPSD.

**Fig. 1:**
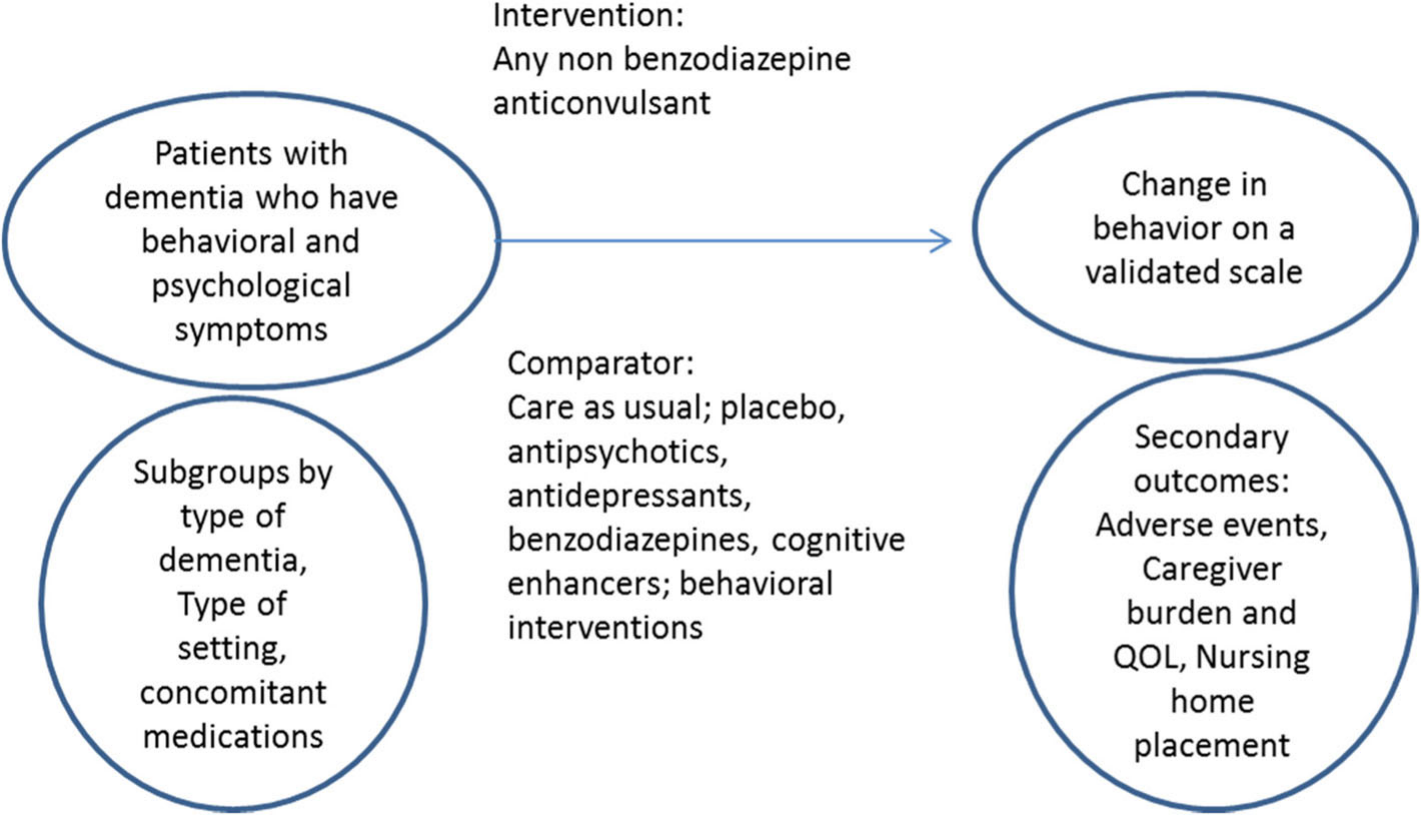
Structure of PICO question

We plan to conduct a systematic review to try to answer the following questions: (Fig. 1).

Key question 1: In patients with dementia who have behavioral and psychological symptoms do anticonvulsants improve patient (decrease in agitation or aggression and nursing home placement) and caregiver (burden and quality of life) outcomes?

Key question 2: In patients with dementia who have behavioral and psychological symptoms do anticonvulsants differ in serious adverse events?

Key question 3: Do treatment effects vary by type of dementia, setting in which the intervention is administered and concomitant pharmacotherapy?

## Methods

This protocol has been registered in the PROSPERO database for systematic reviews, a web-based international registry of systematic review protocols: PROSPERO #CRD42017079826.

A PRISMA-P checklist was completed for this protocol (Additional file 2). Criteria for considering studies for this review were based on the PICOTS format (Table 1).

**Table 1:** Summary or selection criteria

Category	Criteria
Population(s)	Individuals with dementia and BPSD living in the community, long term care, or in specialized longer stay settings
Intervention	All anticonvulsants for BPSD including valproic acid, gabapentin, pregabalin, carbamazepine, phenytoin, topiramate, levetiracetam, zonisamide, oxcarbazepine, lamotrigine, and phenobarbital. Benzodiazepines excluded
Control	Placebo, no intervention, or other active treatments including both non-pharmacologic or pharmacologic treatments
Outcomes	Primary outcomes: We will consider studies that use validated measures of BPSD such as
	1. Neuropsychiatric Inventory [19].
	2. Cohen-Mansfield Agitation Inventory [20]
	3. Brief Psychiatric Rating Scale [21]
	4. Clinical Global Impression Scale
	Secondary outcomes:
	1. Caregiver burden and quality of life
	2. Placement in long term care facility from home.
	3. Serious adverse effects
	4. Treatment discontinuation due to serious adverse effects
Study design	Randomized control trials and crossover trials.
Timing	Any duration of follow-up longer than 2 weeks.

### Eligibility criteria

We will include studies of patients with BPSD residing in long term care facilities, community, or in specialized geriatric assessment and psychogeriatric units. We will exclude studies conducted in acute care hospitals other than psychogeriatric units as the picture in an acute hospital admission is usually confounded by other factors such as delirium. Further, as a majority of BPSD is managed in LTC and the community as a chronic problem, we think that these criteria will capture the studies needed to answer our question. As dementia can occur in younger adults, we did not limit the search to older adults.

The diagnosis of dementia can be arrived at by a clinical interview and exam using criteria specified by the Diagnostic and Statistical Manual of Mental Disorders fourth or fifth editions, or International Statistical Classification of Diseases and Related Health Problems tenth revision (ICD-10), or by using internationally recognized criteria such as National Institute of Neurological and Communicative Disorders and Stroke-Alzheimer’s Disease and Related Disorders Association (NINCDS-ADRDA).

We will include clinical trials of orally administered anticonvulsants, such as valproic acid, gabapentin, pregabalin, carbamazepine, phenytoin, topiramate, levetiracetam, zonisamide, oxcarbazepine, lamotrigine, and phenobarbital without restrictions regarding the type of anticonvulsant, dose, or frequency. We will include studies with both flexible and fixed doses. We will also consider studies in which combinations of anticonvulsants or anticonvulsants plus another drug or non-pharmacologic strategy was the intervention. The interventions will include all non-benzodiazepine anticonvulsants for BPSD. As seizure disorders are a physiologically different condition and the population characteristics of these patients are different, these studies will be excluded.

The control condition for studies can be either placebo, no intervention, or other active treatments including pharmacologic or non-pharmacological interventions. In studies where there is co-administration of other drugs that can impact the outcome such as co-administration of a benzodiazepine, we will only consider trials where the number of patients who received the additional drug does not differ significantly between randomized populations.

We will consider interventions and comparators that allow us to isolate the effect of the anticonvulsant. For example, a comparison between a combination of an anticonvulsant + antidepressant versus an antipsychotic + the same antidepressant may be considered as anticonvulsant vs antipsychotic, whereas a comparison between an anticonvulsant + antidepressant versus just an anticonvulsant would be excluded. We will consider studies that are longer than 2 weeks in length as symptoms lasting less than 2 weeks are more likely to be secondary to delirium. We will include randomized control trials and crossover trials. For crossover studies, we will include pre-crossover, and consider post-cross-over if there is an adequate washout. We defined an adequate washout period as the time required for the intervention drug to reach steady state concentration or five elimination half-lives.

We will include English publications only, but the study may be conducted in any country.

We will include studies that report outcomes based on validated measures of BPSD such as the Neuropsychiatric Inventory [19], Cohen-Mansfield Agitation Inventory [20], Brief Psychiatric Rating Scale [21], and Clinical Global Impression Scale, and these will be considered as primary outcomes.

### Information and searches

Trials will be identified using the following sources: the Cochrane Central Register of Controlled Trials (CENTRAL, latest version), MEDLINE (OVID SP), EMBASE (OVID SP, 1980 to present), PsycInfo (OVID SP), CINAHL (EBSCO), ClinicalTrials.gov, and the World Health Organization International Clinical Trials Registry Platform (WHO ICTRP http://apps.who.int/trialsearch/Default.aspx); ISRCTN will be searched for on-going registered trials. The studies will be limited to those published in English but the study location can be worldwide. The search strategies will be developed by the Clinical Services Librarian Health Sciences Library at McMaster University. Strategies will consist of controlled vocabulary terms and keywords to describe the condition and the intervention and a filter to identify randomized controlled trials. The Boolean operator “OR” will be used to combine terms within each concept and the operator “AND” will be used to combine the concepts together. See Additional file 1 for a draft MEDLINE strategy. The strategy will be replicated as closely as possible across the other databases.

#### Data collection and analysis

Data will be managed using the Covidence software (Covidence systematic review software, Veritas Health Innovation, Melbourne, Australia) throughout the study.

#### Screening and selection of studies

We will screen for duplicate citations. After pilot testing for title exclusion achieves acceptable interindividual agreement between reviewers, two sets of authors will independently screen the citations followed by full text review of trials identified by the literature search. We will resolve disagreements regarding eligibility by consulting with an additional author who is not part of the initial dyad of reviewers.

#### Data collection

Two sets of authors (SB and JT or JH and HA) will independently extract data after piloting the forms with at least two full-text articles to ensure the validity of the forms. Any discrepancies in the extracted data will be resolved by discussion. We will use a standard data extraction form to extract the following information: characteristics of the study (design, method of randomization), participants, setting, interventions, and outcomes (types of outcome measures, serious adverse events). We will then check for accuracy before entering the data into Covidence software.

### Outcomes and prioritization

The primary outcome will be the change in behavior as measured by validated scales. We will also consider studies that present the change in behavior as a dichotomous outcome as long as a validated measurement is used, but these studies will be analyzed separately. Secondary outcomes will include caregiver burden and quality of life and placement in a long-term care facility from home. Other secondary outcomes will include serious adverse effects as defined by FDA or treatment discontinuation due to adverse effect.

We will customize a data extraction tool to extract the following information:

1. The number of patients eligible, number randomized, and reasons why patients were not included in the trial
2. The number of patients evaluated at follow-up(s) and what the follow-up time points were
3. Patient characteristics including age, sex, co-morbidities, diagnosis and type of dementia, type of healthcare or community setting, and stage or severity of disease (for example, as measured by MMSE or CDR)
4. Anticonvulsant used and dose. In studies that use multiple doses of the same medication as different arms, we will pick the arm that reflects current FDA approved dose with modifications allowed for seniors (for example, 20 mg of citalopram and not 10 or 40 mg)
5. Comparison intervention including duration, mode, and dose where applicable
6. Serious adverse events
7. Outcome data at all-time points, including how the outcome was measured and the mean or categorical scores of the main and other outcomes
8. Concurrent use of other drugs including antidepressants, antipsychotics, benzodiazepines, and exclusions
9. Measures of cognition or cognitive decline during or after the intervention, or both
10. Quality of life and caregiver burden and how these were measured
11. Trial design features on masking, whether parallel group or cross-over, features of randomization, and sample size calculation
12. Any necessary additional data on trial design and outcomes to allow assessment of risk of bias. Dropout rates and reasons why. Comment on success of masking, given the possibility of side effects unmasking patients

### Assessment of risk of bias in included studies

For the assessment of study quality, we will follow the guidance of the Cochrane Collaboration [22]. Initially, we will copy information relevant for making a judgment on criteria from the original publication into an assessment table. If additional information is available from study authors, we will also enter this in the table along with an indication that this is unpublished information. Two review authors (JT and JH) will independently make a judgment as to whether the risk of bias for each criteria is considered to be “low,” “unclear,” or “high.” Consensus will be reached with a third author (SB). We will resolve disagreements by discussion. We will consider trials which are classified as low risk of bias in sequence generation, allocation concealment, blinding, incomplete data, and selective outcome reporting as overall low bias risk trials. Blinding of assessors will carry a greater weight in terms or risk of bias as individuals with cognitive impairment may not be as susceptible to the risk of being unblinded, especially in the case of pharmacological intervention.

### Data synthesis

#### Assessment of heterogeneity

We will look for clinical heterogeneity by examining the study details to determine the appropriateness of combining studies quantitatively and, when summary estimates are computed, test for statistical heterogeneity between trial results using the *χ*^2^ test and the *I*^2^ tests statistics when they are combined (see Chapter 9 of *The Cochrane Handbook of Systematic Reviews of Interventions*) [22]. We will classify heterogeneity using the following *I*^2^ values: 0 to 40%, might not be important; 30 to 60%, may represent moderate heterogeneity; 50 to 90%, may represent substantial heterogeneity; and 75 to 100%, considerable heterogeneity. If substantial heterogeneity exists, we will explore reasons for this through sensitivity and subgroup analyses on factors related to risk of bias, study design, and patient and intervention characteristics.

#### Measures of treatment effect

The outcome measures from the individual trials will be combined through meta-analysis when appropriate (based on the clinical comparability of population, intervention, and outcomes between trials) using a random-effects model. A *P* value of less than 0.05, using the *χ*^2^ test, indicates significant statistical heterogeneity. For dichotomous data, we will use relative risk (RR) and absolute risk reduction (ARR) as the effect measures with 95% confidence intervals (CI). For continuous data, we will present the results as mean differences (MD) with 95% confidence intervals (CI). When pooling data across studies, we will estimate the mean difference if the outcomes are measured in the same way between trials. If papers present odds ratios (ORs), then we will try to extract the data, and if not available, then we will contact the authors for datasets.

#### Unit of analysis

The unit of analysis will be the study.

#### Dealing with missing data

We will analyze studies on an intention-to-treat (ITT) basis, i.e., we will analyze patients according to the intervention they were allocated, whether they received the intervention or not. We will impute a poor outcome for a drop-out rate of > 5%. We will also perform a sensitivity analysis imputing a favorable outcome for patients who dropped out from the studies. For each trial, we will report whether or not the investigators stated if the analysis was performed according to the ITT principle.

#### Narrative summary

If a meta-analysis is not possible or appropriate, the results from clinically comparable trials will be described qualitatively in the text. We will narratively describe the major findings and conclusions from individual RCTs. We will identify evidence gaps by documenting conflicting evidence across identified studies and comments when the existing evidence base is insufficient to reach firm conclusions.

### Subgroup analysis and investigation of heterogeneity

In the case of excessive statistical heterogeneity (*I*^2^ > 50%), we will use subgroup analysis to evaluate for potential sources of heterogeneity. Subgroup analyses are secondary analyses in which the participants are divided into groups according to shared characteristics, and outcome analyses are conducted to determine if any significant treatment effect occurs according to that characteristic.

If data permit, we will carry out the following subgroup analyses: (1) effect of intervention by setting such as LTC vs community-dwelling seniors, (2) different types of drug as the associated intervention (e.g., antidepressants versus antipsychotics), (3) types of dementia such as anticonvulsants in Lewy body dementia or Alzheimer’s dementia.

#### Sensitivity analysis

If there are an adequate number of studies, we will perform the following sensitivity analyses:

- Analyze results using last observation carried forward and per protocol analysis
- Analyze data after excluding low-quality studies

#### Assessment of meta-biases

Apart from assessing the risk of selective outcome reporting, considered under assessment of risk of bias in included studies, we will assess the likelihood of potential publication bias using funnel plots, provided that there are at least ten trials [23]. Although small sample effects in a funnel plot can be a marker of publication bias, other causes will be considered including selection biases, poor methodological quality, and heterogeneity, artefactual, and chance. Furthermore, we will contact drug companies and authors also as a strategy to assess reporting bias.

#### Confidence in cumulative estimates

We will use the principles of the GRADE system [24], as recommended in *The Cochrane Handbook for Systematic Reviews of Interventions* to assess the quality of the body of evidence associated with specific outcomes (such as decrease in agitation or aggression, global improvement indices, serious adverse events) in our review and construct a SoF table using the GRADE software GRADEPRO [25]. GRADE specifies an approach to framing questions, choosing outcomes of interest and rating their importance, evaluating the evidence, and incorporating evidence with considerations of values and preferences of patients and society to arrive at recommendations [26]. The main comparison will be anticonvulsants compared to other active treatments including both non-pharmacologic and pharmacologic treatments.

Factors that may decrease the quality of the evidence include risk of bias, imprecision, indirectness, and inconsistency such as when the decision is made on the basis of the variability of results across the included studies. It also takes into account whether all the research evidence has been taken to account or if there is publication bias.

The quality of the evidence for a specific outcome will be reduced by a level, according to the performance of the studies against these five factors. There are five levels of evidence: High-quality evidence: there are consistent findings among at least 75% of RCTs with low risk of bias, consistent, direct, and precise data and no known or suspected publication biases. Further research is unlikely to change either the estimate or our confidence in the results. Moderate quality evidence: one of the domains is not met. Further research is likely to have an important impact on our confidence in the estimate of effect and may change the estimate. Low-quality evidence: two of the domains are not met. Further research is very likely to have an important impact on our confidence in the estimate of effect and is likely to change the estimate. Very low-quality evidence: three of the domains are not met. We are very uncertain about the results. No evidence: no RCTs were identified that addressed this outcome.

## Discussion

Our protocol describes the methods and approach we plan to undertake in systematically reviewing the current evidence for the use of anticonvulsants in the management of behavioral and psychological symptoms of dementia. To the best of our knowledge, this will be the first such systematic review that includes novel anticonvulsants such as topiramate, zonisamide, and levetiracetam for this clinical indication. Many medications that were thought to be initially helpful in the treatment of BPSD have also been shown to cause significant harm. Therefore, we have included serious adverse events as a patient-relevant outcome to our review. With ongoing encouragement from governmental and professional organizations to limit and de-prescribe antipsychotics and benzodiazepines for BPSD in elderly, there is an urgent need to better understand the efficacy of other medication groups that are beginning to be used off label in the management of this very prevalent condition. Some limitations that we anticipate are that adverse events are more likely to be captured in large observational studies and might not be fully captured within this systematic review. We also anticipate that some of the newer anticonvulsants may not have multiple RCTs; however, we will describe these narratively. We will compare our results with those of other systematic reviews that have focused on one medication. While the approach of focusing on one anticonvulsant alone such as valproic acid or carbamazepine provides important clinical information about the utility of the drug, a review that looks at these medications as a group may be able to shed light on whether such findings can be generalized beyond these individual medications. Any major amendments to our protocol will be recorded on PROSPERO.

We hope that our review will be a useful guide to clinicians that help them provide accurate information of about the use of anticonvulsants for behavior management to their patients and caregivers leading to an informed decision-making process for all.

## Additional files

Additional file 1: Preliminary search strategy MEDLINE. (PDF 80 kb)

Additional file 2: PRISMA-P+checklist Anticonvulsant protocol. (DOCX 30 kb)

## Abbreviations

BPSD: Behavioral and psychological symptoms of dementia
GABA: Gamma-aminobutyric acid (γ-aminobutyric acid)
SSRI: Selective serotonin reuptake inhibitors
WHO: World Health Organization
