# Supplementary material for: Anticonvulsants for behavioral and psychological symptoms in dementia: protocol for a systematic review
Source: Syst Rev. 2019 May 18;8:118. doi: 10.1186/s13643-019-1025-5 (PMC6525967; doi:10.1186/s13643-019-1025-5)
Supplement: Supplementary file 1 — Preliminary search strategy MEDLINE. (PDF 80 kb) [file 13643_2019_1025_MOESM1_ESM.pdf]

Database: OVID Medline Epub Ahead of Print, In-Process & Other Non-Indexed Citations, Ovid MEDLINE(R) Daily and Ovid MEDLINE(R) 1946 to Present
Search Strategy:
----
1 exp Dementia/ (144033)
2 dement*.ti,ab,kf. (96495)
3 alzheimer*.ti,ab,kf. (123728)
4 huntington*.ti,ab,kf. (15746)
5 lewy bod*.ti,ab,kf. (7811)
6 (cognit* adj2 (declin* or deficit* or impair*)).ti,ab,kf. (85446)
7 (creutzfeldt* or binswanger* or korsakoff* or wernicke*).ti,ab,kf. (10630)
8 or/1-7 (280873)
9 exp Anticonvulsants/ (130268)
10 (anticonvuls* or antiepileptic*).ti,ab,kf. (42216)
11 Acetazolamide*.ti,ab,kf. (6665)
12 Bromide*.ti,ab,kf. (49264)
13 Carbamazepine*.ti,ab,kf. (13701)
14 Chlormethiazole.ti,ab,kf. (442)
15 Clonazepam*.ti,ab,kf. (3425)
16 Clorazepate Dipotassium.ti,ab,kf. (71)
17 Diazepam*.ti,ab,kf. (19071)
18 Dimethadione*.ti,ab,kf. (101)
19 Estazolam*.ti,ab,kf. (191)
20 Ethosuximide*.ti,ab,kf. (1194)
21 Flunarizine*.ti,ab,kf. (1588)
22 Lorazepam*.ti,ab,kf. (3480)
23 Magnesium Sulfate*.ti,ab,kf. (3491)
24 Medazepam*.ti,ab,kf. (216)
25 Mephenytoin*.ti,ab,kf. (845)
26 Mephobarbital*.ti,ab,kf. (89)
27 Meprobamate*.ti,ab,kf. (1788)
28 Nitrazepam*.ti,ab,kf. (976)
29 Paraldehyde*.ti,ab,kf. (372)
30 Phenobarbital*.ti,ab,kf. (16643)
31 Phenytoin*.ti,ab,kf. (11142)
32 Pregabalin*.ti,ab,kf. (2746)
33 Primidone*.ti,ab,kf. (1134)
34 Riluzole*.ti,ab,kf. (1312)
35 Thiopental*.ti,ab,kf. (4949)
36 Tiletamine*.ti,ab,kf. (344)
37 Trimethadione*.ti,ab,kf. (353)
38 Valproic Acid*.ti,ab,kf. (7624)
39 Vigabatrin*.ti,ab,kf. (1828)
40 Gabapentin*.ti,ab,kf. (5517)
41 Topiramate*.ti,ab,kf. (4133)
42 Levetiracetam*.ti,ab,kf. (3022)
43 Zonisamine*.ti,ab,kf. (0)
44 Oxcarbazepine*.ti,ab,kf. (1733)
45 (clobazam or lacosamide or rufinamide or stiripentol or divalproex or felbamate or tiagabine or brivaracetam or eslicarbazepine or ethotoin or trimethadione).ti,ab,kf. (4471)
46 Trimethadione/ (487)
47 or/9-46 (221033)
48 8 and 47 (3256)
49 Psychomotor Agitation/ (4671)
50 exp Aggression/ (34091)
51 (agitat* or aggress* or hitting or kicking or grabbing or pushing or throwing or biting or scratching or spitting or hurting or wandering or falling or hoarding or

restless* or screaming or cursing or complaining).ti,ab,kf. (262345)
52 49 or 50 or 51 (278276)
53 48 and 52 (332)

****

1.
Sequential drug treatment algorithm for agitation and aggression in Alzheimer's and mixed dementia.
Davies SJ; Burhan AM; Kim D; Gerretsen P; Graff-Guerrero A; Woo VL; Kumar S; Colman S; Pollock BG; Mulsant BH; Rajji TK.
Journal of Psychopharmacology. 269881117744996, 2018 Jan 01.
[Journal Article]
UI: 29338602
Authors Full Name
Davies, Simon Jc; Burhan, Amer M; Kim, Donna; Gerretsen, Philip; Graff-Guerrero, Ariel; Woo, Vincent L; Kumar, Sanjeev; Colman, Sarah; Pollock, Bruce G; Mulsant, Benoit H; Rajji, Tarek K.
